# HDTMS-, Polybutadiene-, and Benzotriazole-Modified Polylactic-Based Resin for Solar Cells Encapsulation with Exceptional Environmental Stability of MAPI Perovskite Films

**DOI:** 10.3390/molecules31030427

**Published:** 2026-01-26

**Authors:** Ayad Aicha Aziza, Elbar Mohamed, Ievgen Zaitsev, Kuchansky Vladislav

**Affiliations:** 1 Materials Science and Informatics Laboratory, Faculty of Science, University of Djelfa, Djelfa 17000, Algeria; ayadaziza120@gmail.com; 2 Applied Automation and Industrial Diagnostics Laboratory (LAADI), Faculty of Science and Technology, Ziane Achour University, Djelfa 17000, Algeria; m.elbar@univ-djelfa.dz; 3 Department of Theoretical Electrical Engineering and Diagnostics of Electrical Equipment, Institute of Electrodynamics, National Academy of Sciences of Ukraine, Peremogy, 56, 03057 Kyiv, Ukraine; 4 Department of Power-Supply Systems Optimization, Institute of Electrodynamics, National Academy of Sciences of Ukraine, Beresteyskiy, 56, 03057 Kyiv, Ukraine; kuchanskiyvladislav@gmail.com

**Keywords:** perovskite, stability, MAPI, HDTMS, EPB, benzotriazole, polylactic-based resin (PLA), solar cells, encapsulation

## Abstract

In this work, we report a protective encapsulation intended as the final coating layer on solar cells. The formulation consists of polylactic (PLA)-based resin, modified with hexadecyltrimethoxysilane (HDTMS), epoxidized polybutadiene (EPB), and benzotriazole as a UV absorber with approximate weight fractions ranging from 20 to 60 wt% for PLA, 30–80 wt% for solvents (toluene and chloroform), and 0–5 wt% for HDTM, EPB, and benzotriazole with percentages 54.2%, 29.2%, and 16.7%, respectively. The encapsulating material, due to its insulating nature and high optical transparency, surpasses that of ethylene–vinyl acetate (EVA), as demonstrated in this study. To assess the protective effect of the developed formulation, the study focused on applying the modified PLA resin onto isolated methylammonium lead iodide (MAPI) perovskite films on glass substrates. The samples were prepared as isolated MAPI absorbers to specifically assess the intrinsic contribution of the dual encapsulation configuration at its real position in a complete solar cell stack, demonstrating that even this unoptimized perovskite film exhibits remarkable stability and excellent structural and optical retention over two months under the protective scheme (86% of its initial structural stability, as quantified from integrated XRD peak intensities, and 68% of its initial optical absorbance, determined from the integrated UV–Vis spectra), whereas the uncoated films showed significant degradation. Although MAPI was selected as a model system due to its well-known environmental instability, the proposed encapsulation material and methodology are not limited to this architecture and can, in principle, be applied to various photovoltaic technologies. These findings demonstrate the strong potential of the polylactic-based resin as an effective environmental barrier for solar cells and provide a solid foundation for future full-device integration studies.

## 1. Introduction

Solar panel encapsulation is an important step to protect panels against the environmental conditions. Ethylene–vinyl acetate polymer (EVA), polyurethane (PU), and epoxy films based on resins are usually used to protect solar panels, providing a barrier structure to restrict the penetration of oxygen and moisture. Usually, solar cell encapsulation is applied as a film via a lamination process that consists of three main steps: heating the encapsulant homogeneously to ensure homogeneous curing of the encapsulant, applying vacuum to remove air bubbles and unwanted impurities, and applying pressure to ensure proper surface contact and adhesion between the encapsulant and other parts of the solar cell. These three steps are carried out at high temperature and pressure, which may affect the properties of the solar cell and lead to its degradation [1].

The third generation of solar cells saw the appearance of perovskite cells (PSCs) [2]. The research has continued thereafter to solve many problems of these PSCs such as improving efficiency, stability, cost, etc. Perovskite materials used in solar cells can be broadly categorized based on their cation composition. They include fully inorganic perovskites (e.g., CsPbI_3_ and CsPbBr_3_) and hybrid organic–inorganic perovskites with the general formula ABX_3_, where A is an organic cation, B is a metal cation, and X is a halide. Among these, CH_3_NH_3_PbI_3_ (MAPI) is considered one of the most stable and best-performing absorbers, primarily because the lead (Pb^2+^) cation provides higher intrinsic stability compared to tin-based (Sn^2+^) analogs (e.g., CH_3_NH_3_SnI_3_), which are more prone to oxidation and degradation under environmental stress. In perovskite solar cell devices, MAPI is typically implemented as an intrinsic absorber layer, meaning it is used without intentional p-type or n-type doping. The general architectures for solar cells based on this intrinsic layer include p-type/intrinsic/n-type (PIN) and n-type/intrinsic/p-type (NIP) configurations, and each of these architectures can be realized in either planar or mesoporous designs. In these devices, the hole transport layer (HTL) extracts and transports positive charges, while the electron transport layer (ETL) extracts and transports electrons, facilitating efficient charge separation and collection [3,4,5,6]. Despite the high efficiency of this type of perovskite, the instability remains a big problem to be faced. The degradation of the perovskite material in terms of aging under environmental conditions such as oxygen, humidity, high temperatures, and UV rays affects their performance, degrading the perovskite layer by removing its organic component and retaining only the inorganic element (PbI_2_) [7,8,9,10,11,12,13,14,15,16]. Until now, this problem had not yet been resolved, despite the many efforts undertaken. We note that the results of different works have only led to the improvement of the duration of the stability of the perovskite [17,18,19,20].

The encapsulation concept of PSC devices is still premature and is based mainly on materials possessing a low water vapor/oxygen transmission rate, thermal stability up to 85 °C, low temperature (≤120 °C) to ensure compatibility with the thermal stability of perovskite, optical transparency (transmittance ≥ 90% from 400 to 1100 nm) for front-side encapsulants, and mechanical properties such as adhesion strength to withstand thermos-mechanical stresses originated from daily temperature variation [21,22]. A recent study reported an effective encapsulation strategy based on a self-crosslinked fluorosilicone polymer gel, achieving non-destructive encapsulation at room temperature by using an unspecified epoxy edge sealant [23]. Encapsulation by high-throughput methodologies has rarely been described, and aging tests have not been systematically carried out [24]. These well-established encapsulation strategies cannot meet the distinctive requirements of PSCs. Starting from these problems, the idea of the present work arose in which the conditions are fulfilled. Remember that our samples were studied under weather conditions for a period of two full months. The encapsulation is usually the last step to be performed on the photovoltaic panels. In this work, we perform preliminary stability tests of the MAPI absorber layer under real weather conditions by studying the properties under encapsulation and evaluating how the HDTMS-modified polylactic resin (HDTMS-modified PLA) protective layer affects device lifetime. Our contribution lies in providing a solution to stabilize the MAPI structure while maintaining acceptable properties for an extended period. In addition, we employed spin coating as a simple and inexpensive method for encapsulation. This technique avoids undesirable factors such as elevated temperature and mechanical stress. The HDTMS-modified PLA used in this work is a hydrophobic material with high optical transparency [25,26]. Pristine PLA has not been previously used as an encapsulation material for perovskite solar cells. Instead, earlier studies employed PLA exclusively for interfacial engineering or as a minor micro-incorporated component within the device structure [27,28], without reporting any stability or aging tests. This limited use is mainly attributed to the intrinsic drawbacks of pristine PLA, including pronounced brittleness and high susceptibility to hydrolytic degradation [29]. Moreover, due to its electrically insulating nature, PLA can adversely affect charge transport if applied in significant thicknesses, which restricts its utilization for ultrathin interfacial layers. In the present work, these limitations are addressed through deliberate material modification: HDTMS is introduced to enhance resistance against hydrolytic degradation, polybutadiene is incorporated to overcome brittleness and impart mechanical flexibility, and benzotriazole is added to improve resistance against UV-induced photodegradation. Following these modifications, the resulting material is evaluated for the first time as an external encapsulation layer by applying it onto perovskite films to assess its protective effectiveness against environmental stressors.

## 2. Results

### 2.1. HDTMS-Modified PLA Characterization

**Figure 1:**
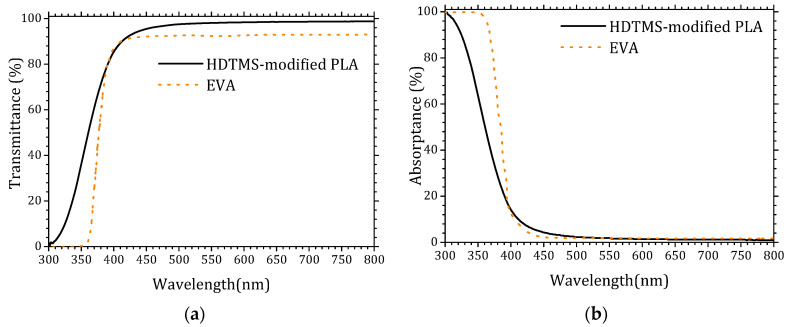
UV–visible spectra of the HDTMS-modified PLA and EVA. (**a**) Transmittance and (**b**) absorptance.

Encapsulation materials typically exhibit high optical transmittance, often approaching that of glass substrates (~100%). However, their intrinsic chemical composition usually introduces a non-negligible absorption component that reduces the effective photon flux reaching the solar cell. Figure 1 presents a comparative evaluation of the transmittance (a) and absorptance (b) spectra of the HDTMS-modified PLA used in this study and the widely employed EVA encapsulant. The transmittance spectrum of EVA was extracted from reference [30], while its absorptance spectrum was obtained from reference [31].

The optical characterization of the HDTMS-modified PLA demonstrates its excellent suitability as an encapsulation material for photovoltaic devices. The HDTMS-modified PLA exhibits a significantly higher transmittance in the UV–visible range compared to conventional EVA, particularly between 300 and 400 nm, where most photovoltaic absorbers benefit from increased photon flux. Conversely, EVA shows noticeably stronger UV absorption, which limits the availability of high-energy photons reaching the active layer. The reduced absorptance and enhanced UV transmittance of the HDTMS-modified PLA indicate a lower optical loss mechanism, confirming its advantage as a transparent encapsulant capable in maximizing incident irradiance on the solar cell. These results highlight the high optical quality of HDTMS-modified PLA and its potential as a promising alternative to traditional EVA encapsulation. The detailed data are provided in the Supplementary Materials (Tables S1 and S2).

**Figure 2:**
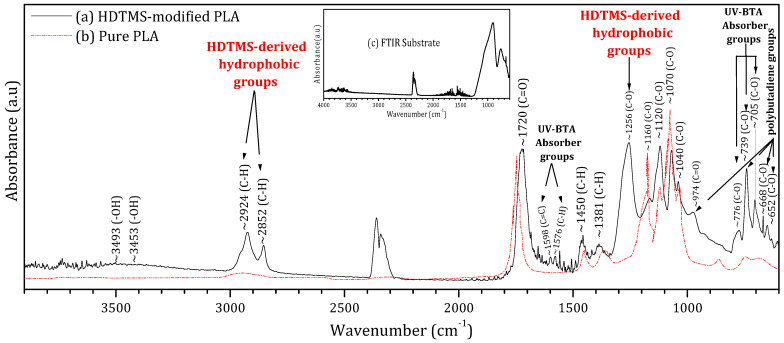
Fourier transform infrared spectroscopy (FTIR) of the (**a**) HDTMS-modified PLA deposited on a glass substrate, (**b**) pure PLA, and (**c**) glass substrate.

To assess its chemical composition and quantify the fraction of pure PLA within the formulation, Figure 2 presents the FTIR spectrum of the deposited HDTMS-modified PLA (black curve) alongside the reference spectrum of pure PLA (red curve) extracted from reference [32]. This comparison aims to quantify the fraction of pure PLA within the commercial HDTMS-modified PLA as well as the additional vibrational signatures arising from the incorporated additives.

The superposition of both spectra shows that the main fingerprint peaks of pure PLA are clearly preserved within the HDTMS-modified PLA. These characteristic PLA bands occur at 1040, 1070, 1120, and 1160 cm^−1^ (C–O stretching vibrations of the ester backbone); 1381 and 1450 cm^−1^ (C–H bending modes); and 1720 cm^−1^ (the strong carbonyl stretching (C=O) characteristic of PLA [32,33]); and 2852 and 2924 cm^−1^ (C–H stretching vibrations in the polymer chains [32,33,34,35]). By integrating the areas of these PLA-specific peaks relative to the overall spectrum, the proportion of pure PLA in the HDTMS-modified PLA was estimated to be approximately 76%.

Based on the literature, the additional peaks observed in the FTIR spectrum of the HDTMS-modified PLA can be attributed to the three types of additive components (HDTMS, polybutadiene, and benzotriazole).

Moisture-resistant additives are commonly introduced into polymeric encapsulants to enhance environmental stability and suppress hydrolytic degradation [33]. In the present HDTMS-modified PLA, the FTIR band observed at ~1256 cm^−1^ can be assigned to C–O–Si/Si–O–C stretching vibrations associated with hexadecyltrimethoxysilane (HDTMS). In addition, absorption bands located at ~2924 and 2852 cm^−1^, corresponding to C–H stretching modes of long aliphatic chains, are present in both pure PLA and HDTMS-containing systems [36,37,38]. However, their significantly higher intensity in the HDTMS-modified PLA compared to pure PLA indicates an increased contribution from long alkyl chains introduced by HDTMS. This enhanced aliphatic content is consistent with the formation of a hydrophobic, moisture-barrier surface layer, which effectively limits water ingress and improves the environmental durability of the encapsulated perovskite films. UV-absorbing additives are commonly incorporated into encapsulation materials to protect them from photodegradation under prolonged light exposure. However, their presence can induce additional absorption in the UV region, thereby reducing the number of incident photons reaching the active layer. In the present formulation, 2-(2-hydroxyphenyl)-benzotriazole (UV-BTA) was employed for this purpose. Its incorporation is evidenced by the characteristic FTIR bands at 705, 739, and 776 cm^−1^ (C–O); 1576 cm^−1^ (C–H); and 1598 cm^−1^ (C=C) [39,40]. Notably, the use of benzotriazole resulted in significantly reduced UV absorption compared to conventional EVA encapsulants, with a much weaker impact on photon transmission, as confirmed by the UV–visible measurements. Mechanical-property modifiers are commonly incorporated to reduce the intrinsic brittleness and low toughness of pure PLA [27]. In the present formulation, polybutadiene was employed for this purpose. Its presence is associated with the additional absorption bands observed at 652, 668, and 739 cm^−1^ (C–O) and 974 cm^−1^ (C=O) vibrational modes [41,42,43]. These additives improve the rigidity, ductility, and thermal stability of the resin, enhancing its suitability for outdoor encapsulation applications. Moreover, the weak broad band detected around 3453–3493 cm^−1^, attributed to O–H hydrogen bond vibrations, may arise from interactions between PLA and these mechanical modifiers. The detailed data are provided in the Supplementary Materials (Table S3).

### 2.2. Structural Characterization

**Figure 3:**
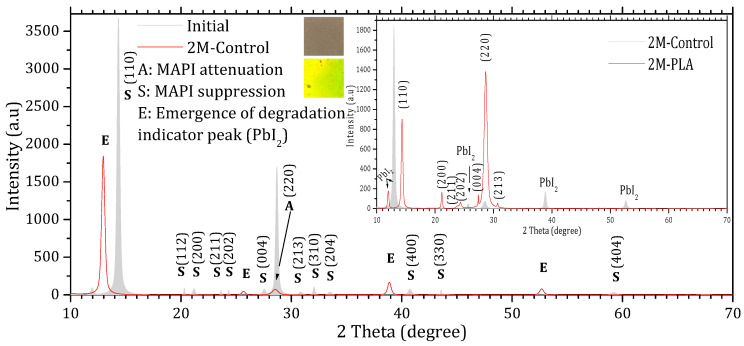
X-ray diffraction (XRD) patterns of MAPI samples without HDTMS-modified PLA encapsulation. The gray diffractogram corresponds to the initial analysis, while the red diffractogram represents the measurements after 2 months of exposure.

**Figure 4:**
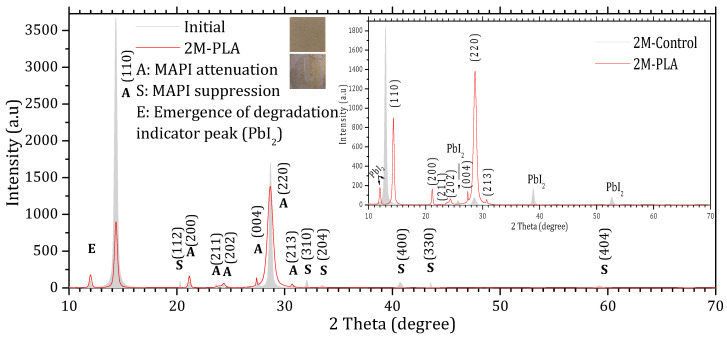
X-ray diffraction (XRD) patterns of MAPI samples with HDTMS-modified PLA encapsulation. The gray diffractogram corresponds to the initial analysis, while the red diffractogram represents the measurements after 2 months of exposure.

Figure 3 and Figure 4 show the XRD patterns of the samples’ initial analysis (Initial), after two months of exposure without encapsulation (2M-control) and with encapsulation (2M-PLA). The diffraction lines characteristic of the MAPI phase in the initial measurement are observed around 2θ ≈ 14.33°, 20.30°, 21.18°, 23.64°, 24.31°, 27.53°, 28.68°, 30.84°, 32.06°, 33.51°, 40.73°, 43.56°, and 59.21°, corresponding, respectively, to the (110), (112), (200), (211), (202), (004), (220), (213), (310), (204), (400), (330), and (440) planes. According to JCPDS card No. 96-451-8044, the film is polycrystalline in nature with a tetragonal structure. An additional line located at 11.94° is attributed to the PbI_2_ phase (JCPDS card No. 01-080-1000).

Comparing the XRD patterns of Initial and 2M-control (Figure 3), the perovskite phase undergoes a complete transformation into the lead iodide (PbI_2_) phase. This transformation is evidenced by the suppression of all characteristic MAPI peaks, except for the one at 28.68°, and the emergence of new diffraction peaks characteristic of PbI_2_, notably at 25.70°, ~38.97°, and ~52.64°, which become clearly visible after two months of exposure. In addition, the original MAPI peak located at 11.97° shifts toward 12.97°, accompanied by a significant increase in its intensity. According to ref. [44], such a shift is typically associated with lattice strain induced by prolonged exposure to environmental stressors (temperature, humidity, and light), which alters the structural integrity of the perovskite lattice. The peak shift is minimal in the presence of HDTMS-modified PLA (11.97°), highlighting its effective role in mitigating lattice strain. The sample color also changes from the characteristic gray of MAPI to the yellow of PbI_2_. In contrast, the MAPI sample encapsulated with HDTMS-modified PLA (Figure 4) exhibits greater structural stability. Only slight attenuation is observed in the two main peaks at 14.33° and 28.68°, along with the suppression of minor peaks corresponding to the (112), (310), (204), (400), (330), and (404) planes. This indicates minor structural deterioration, with no visible change in the sample’s color. It is worth noting that in both samples, the strongest line at 14.33° is noticeably affected more rapidly than the weaker line at 28.68° during degradation. This degradation pattern has been reported in ref. [45] and is attributed to surface decomposition with the loss of organic cations and the formation of PbI_2_, which reduces the coherent scattering volume of the perovskite phase. Since the (110) peak is the first and most intense in MAPI, it is the most sensitive to any reduction in crystal size, so surface degradation affects it before other peaks such as (220) at 28.68°.

The calculation of the texture coefficient R (hkl) revealed that the (110) plane exhibits the highest preferred orientation among all characteristic MAPI reflections. Specifically, the diffraction peak located at 2θ ≈ 14.33°, corresponding to the (110) plane, showed the largest texture coefficient ≈ 61.11%, confirming that this plane not only represents the most intense reflection in the XRD pattern but also constitutes the dominant growth orientation within the MAPI perovskite layer. The detailed data are provided in the Supplementary Materials (Tables S4 and S5). The XRD peak parameters are presented in Table 1, while the corresponding structural properties derived from these data are summarized in Table 2.

**Figure 5:**
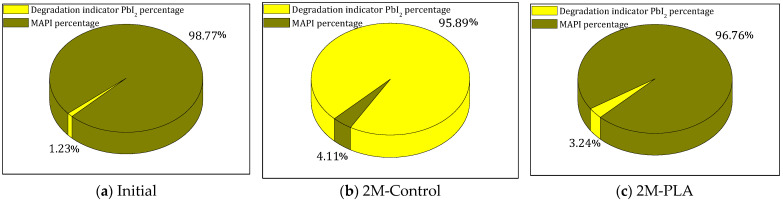
Comparison of MAPI and PbI_2_ phases for the three studied cases.

The evolution of the phase composition of the MAPI samples is illustrated in Figure 5 by three pie charts corresponding to the initial state, the 2-month control (2M-control), and the encapsulated sample (2M-PLA). In the initial analysis, MAPI represents 99% of the crystalline composition. After 2 months of environmental exposure without encapsulation (2M-control), a severe degradation process occurs, resulting in a drastic increase in the PbI_2_ degradation phase to 96%, while the remaining perovskite fraction drops to only 4%. In contrast, the encapsulated sample (2M-PLA) maintains a high level of structural integrity, retaining 97% of the MAPI phase, with only a minimal formation of PbI_2_. This comparison clearly demonstrates the protective effect of HDTMS-modified PLA encapsulation, which significantly mitigates phase decomposition under ambient conditions. The detailed data are provided in the Supplementary Materials (Tables S6–S8).

**Figure 6:**
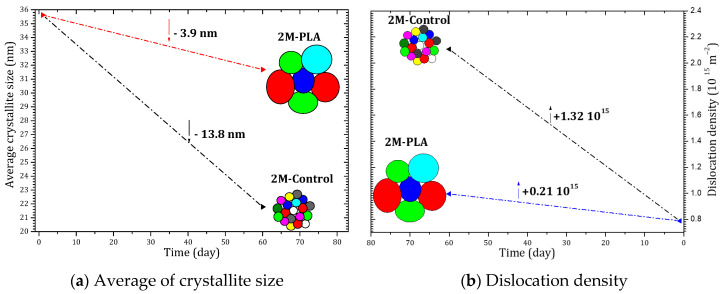
Evolution of crystallite size and dislocation density of MAPI with (2M-PLA) and without (2M-control) PLA encapsulation over 2 months of environmental exposure.

Figure 6 summarizes the temporal evolution of the average crystallite size and the dislocation density for the MAPI samples with and without encapsulation. As shown in Figure 6a, the unprotected sample (2M-Control) undergoes a pronounced reduction in crystallite size, decreasing by 13.8 nm after 2 months of environmental exposure. This significant grain size shrinkage reflects the progressive structural collapse associated with the formation of PbI_2_. In contrast, the encapsulated sample exhibits only a minimal reduction of 3.9 nm, indicating that the polymer layer effectively limits microstructural degradation and preserves the crystalline coherence of the perovskite domains.

A similar trend is observed in the evolution of the dislocation density, shown in Figure 6b. The non-encapsulated sample displays a substantial increase of 1.32 × 10^15^ lines/cm^2^, confirming the accumulation of structural defects and lattice distortion during aging. However, when encapsulation is applied, the increase is drastically reduced to only 0.21 × 10^15^ lines/cm^2^, demonstrating that the protective layer mitigates defect formation and stabilizes the perovskite lattice over time.

Overall, the comparative analysis clearly demonstrates that encapsulation significantly suppresses both crystallite size deterioration and defect density accumulation, thereby enhancing the structural stability of MAPI under ambient aging conditions. The detailed data are provided in the Supplementary Materials (Tables S9 and S10).

### 2.3. Optical Characterization

#### 2.3.1. Absorbance

**Figure 7:**
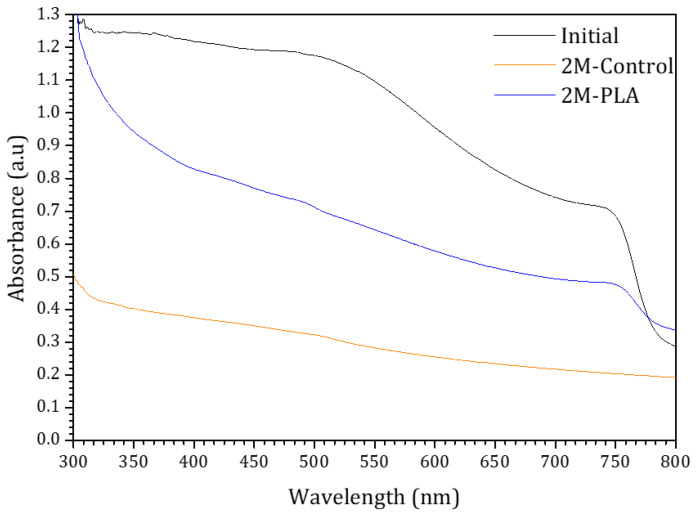
The absorbance spectra of the samples for initial analysis and (without/with) HDTMS-modified PLA after two months of exposure.

To evaluate the effect of HDTMS-modified PLA encapsulation on the absorbance behavior, the initial absorbance spectrum of the sample, along with its temporal evolution under environmental exposure, is presented in Figure 7. The blue and orange curves correspond to the encapsulated and non-encapsulated samples, respectively. For the initial analysis of MAPI, strong absorbance is observed in the 300–520 nm range, in agreement with findings reported in ref. [46]. Compared to the encapsulated sample, the absorbance of the non-encapsulated sample decreases markedly over 2 months, becoming significantly lower across the entire spectral range. The curvature observed at ~780 nm is attributed to the perovskite phase, as suggested by ref. [47]. This feature progressively diminishes as the perovskite degrades in the non-encapsulated sample, whereas it remains clearly detectable in the encapsulated sample, demonstrating the effectiveness of HDTMS-modified PLA encapsulation in preserving the optical stability of the perovskite film over time. The detailed data are provided in the Supplementary Materials (Table S11).

#### 2.3.2. Gap Energy

**Figure 8:**
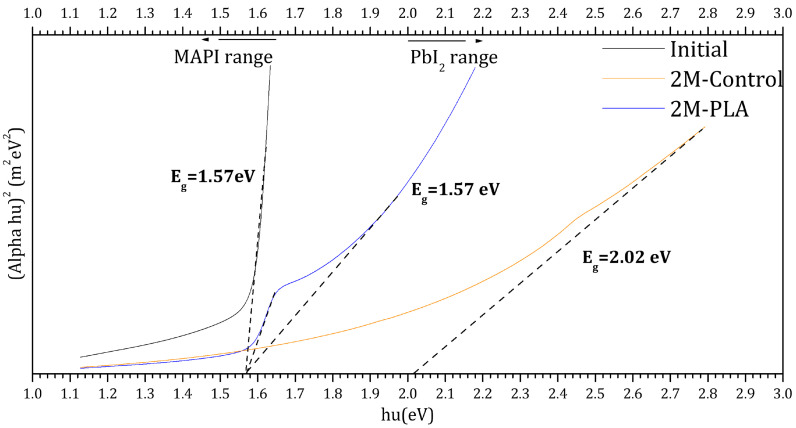
Tauc plots for the samples for initial analysis and (without/with) HDTMS-modified PLA after two months of exposure.

The Tauc plots of the samples are presented in Figure 8. The black curve corresponds to the initial analysis of MAPI, exhibiting a direct optical band gap of 1.57 eV. This value falls within the typical range reported for MAPI perovskite in refs. [48,49,50,51]. After two months of environmental exposure, the encapsulated sample (blue curve) maintained the same band gap value (1.57 eV), indicating excellent stability of the perovskite phase under HDTMS-modified PLA protection. In contrast, the non-encapsulated sample (red curve) exhibited a significant shift in the band gap to 2.02 eV, which is attributed to the formation of PbI_2_ as a degradation product [52]. These results clearly demonstrate the strong protective effect of the HDTMS-modified PLA layer in preserving the structural and optical integrity of the MAPI film over time. The detailed data are provided in the Supplementary Materials (Table S12).

### 2.4. Result Interpretation

In hybrid organic–inorganic halide perovskites such as CH_3_NH_3_PbI_3_, environmental humidity represents a major threat to the structural integrity of the perovskite lattice. Water molecules can readily penetrate the film, triggering hydration processes and subsequent decomposition into hydrated intermediates or into PbI_2_ accompanied by volatile by-products such as CH_3_NH_3_I, CH_3_NH_2_, and HI (Equations (1) and (2)) [53,54]. This moisture-induced degradation progressively disrupts the [PbI_6_]^4−^ octahedral framework, which is essential for maintaining the characteristic optoelectronic properties of the perovskite material [55].

(1) $$2\;{\mathrm{CH}}_{3}{\mathrm{NH}}_{3}{\mathrm{PbI}}_{3}\;(s)\;+\;O_{2}\;(g)\;\overset{O_{2}}{\to }\;2\;{\mathrm{PbI}}_{2}\;(s)\;+\;I_{2}\;(g)\;+\;2\;{\mathrm{CH}}_{3}{\mathrm{NH}}_{2}\;(g)\;+\;2\;\mathrm{HI}\;(g)$$

(2) $$4\;{\mathrm{CH}}_{3}{\mathrm{NH}}_{3}{\mathrm{PbI}}_{3}\;(s)\;+\;2\;H_{2}O\;(\mathrm{lq})\;\overset{H_{2}O}{\to }4\;{\mathrm{CH}}_{3}{\mathrm{NH}}_{3}I\;(g)\;↑\;+\;4\;{\mathrm{PbI}}_{2}\;(s)\;+\;2\;H_{2}O\;(\mathrm{lq})$$

In addition to moisture and oxygen exposure, the intrinsic instability of MAPI under illumination and elevated temperatures plays a critical role in determining its degradation behavior, particularly when comparing non-encapsulated and encapsulated films. Previous studies have demonstrated that photo-irradiation accelerates the formation of iodine vacancies and promotes ionic migration within the perovskite lattice. These processes facilitate phase segregation and enhance the release of volatile decomposition products [56]. Thermal stress further destabilizes the [PbI_6_]^4−^ octahedral network by inducing lattice expansion and accelerating the transformation of MAPI into PbI_2_, especially at temperatures above 85 °C, where thermally driven deprotonation of MA^+^ becomes significant [57]. Moreover, recent reports indicate that the combined effects of heat and light lead to accelerated halide loss and irreversible structural collapse, ultimately degrading the optical and electronic quality of perovskite films [58]. Applying a HDTMS-modified PLA as a capping layer appears to significantly retard these degradation pathways. The dominant stabilization mechanism is physical encapsulation: HDTMS-modified PLA forms a continuous, pinhole-free, and moisture-resistant (HDTMS) coating over the perovskite surface, effectively acting as a barrier against water and oxygen ingress. This encapsulation also limits the escape of volatile species and suppresses thermally activated reactions that would otherwise accelerate degradation. Such an external encapsulation strategy is consistent with well-established stabilization approaches in perovskite solar cell research, where barrier layers are among the most effective extrinsic methods for enhancing environmental stability against humidity [59]. Beyond simple physical protection, the chemical functionalities of PLA, particularly its terminal hydroxyl (–OH) and ester carbonyl (C=O) groups, may also contribute to improved stability through weak interfacial interactions. Hydrogen bonding or dipole–dipole interactions between these polar groups and surface species of the perovskite (such as methylammonium cations or Pb–I units) can partially passivate surface defects and vacancy sites that typically facilitate moisture-induced degradation. This interpretation aligns with recent studies demonstrating that polymeric or organic surface coatings can significantly enhance perovskite stability by both restricting water access and passivating surface states [60]. Consequently, the effective preservation of the structural and likely optoelectronic properties observed upon the application of HDTMS-modified PLA can be attributed to a synergistic stabilization effect involving (i) physical blocking of water and oxygen infiltration, (ii) weak chemical interactions at the interface that reduce surface reactivity, and (iii) suppression of ion migration and phase transitions that drive degradation. Given that, even under dry conditions, the intrinsic thermodynamic nature of CH_3_NH_3_PbI_3_ favors decomposition into CH_3_NH_3_I and PbI_2_ according to first-principle analyses, and the protective role of HDTMS-modified PLA becomes particularly critical for ensuring long-term material stability [61].

## 3. Materials and Methods

### 3.1. Materials

#### 3.1.1. Our Elaboration

**Figure 9:**
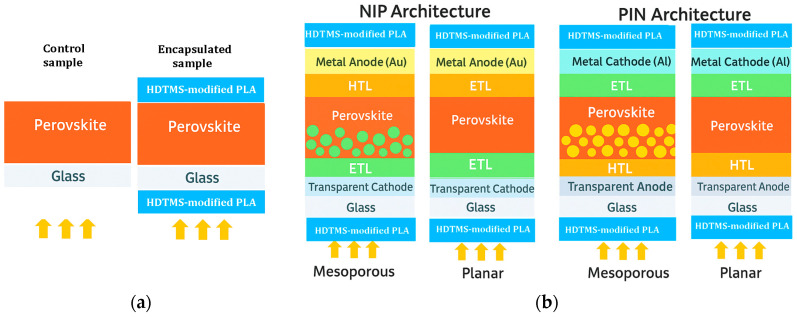
Overview: (**a**) Experimental MAPI films prepared in this work without and with the HDTMS-modified PLA encapsulation; (**b**) schematic illustration of the four commonly used perovskite solar cell architectures (NIP, PIN, mesoporous, and inverted), along with the typical placement of the encapsulation layer. The arrows represent the incident solar light.

The deposition of the samples went through several steps, starting with the preparation of the CH_3_NH_3_PbI_3_ matrix until the film’s deposition. First, methylammonium iodide (MAI) powder was prepared in an ice bath at ~0 °C, and 27.8 mL of methylamine (40 wt% in methanol, TCI) was mixed with 30 mL of hydroiodic acid (57 wt% water) dropwise for 2 h. The solution was evaporated at 50 °C for 1 h by using a rotary evaporator (Rotavap) The precipitate was washed thoroughly with diethyl ether and dried in a low-temperature oven following the same procedure as that reported in ref. [62]. Second, regarding the preparation of the inorganic element (PbI_2_), a powder was prepared by separately dissolving 0.8 g of lead nitrate (Pb (NO_3_)_2_) and 1.0 g of potassium iodide (KI) in 100 mL of distilled water for 15 min, and then these two solutions were mixed dropwise with the addition of 300 mL of distilled water. The final solution was heated for 30 min at 70 °C and then it was poured into a filter paper after cooling. The precipitate was dried at 60 °C for 1 h, and a yellow powder was obtained in accordance with ref. [63]. The third step is the preparation of MAPI (CH_3_NH_3_PbI_3_) perovskite solution: in a flask, equimolar powder of CH_3_NH_3_I and PbI_2_ was mixed in a ratio (1:4) of N,N-dimethyl formamide (DMF) and dimethyl sulfoxide (DMSO), and the solution was agitatedly filled at 60 °C for an entire night. Finally, the polylactic acid-based resin and perovskite thin film were deposited by spin coating on cleaned soda lime substrates (20 mm × 20 mm × 2 mm) under normal conditions at ambient air and room temperature. For the MAPI perovskite layer, it was spun at 2000 rpm for 20 s and then dried at 70 °C for 15 min. This sample was cut with a diamond pen into two parts: the first serves as a reference for the study, and the second will be encapsulated. For the encapsulated device, 0.1 mL of HDTMS-modified PLA solution was deposited by spin-coating on the MAPI layer dropwise at 5000 rpm for 60 s and was dried for 24 h at room temperature. After drying, a second HDTMS-modified PLA layer was deposited onto the sun-facing side of the substrate, serving as the external encapsulant, which typically acts as the optical window in full solar cell architectures. These conditions resulted in an approximate thickness of 800 nm for MAPI and about 0.3 mm for the encapsulation layer, which lies comfortably within the commonly accepted range of 0.2–0.5 mm for standard encapsulant layers. Figure 9a illustrates the experimental MAPI films prepared in this work without and with the HDTMS-modified PLA encapsulation and Figure 9b shows the schematic illustration of the four commonly used perovskite solar cell architectures (NIP, PIN, mesoporous, and inverted), along with the typical placement of the encapsulation layer.

#### 3.1.2. HDTMS-Modified PLA Resin Synthesis: Formulation Following Manufacturer Guidelines and Literature Data

According to the manufacturer, the resin was prepared following a conventional coating formulation process. The formulation is based on polymeric binders dissolved in common organic and aromatic solvents, combined with functional additives through successive wetting and dispersion steps to ensure complete homogeneity. In this context, the main constituents approximately correspond to 20–60 wt% polymeric binder, 30–80 wt% solvents, and ~5 wt% additives, summing to 100% of the formulation, which reflects the overall design of the industrial coating system. Given that our system is based on PLA, as confirmed by FTIR analysis, the preparation was adapted from the established polymer-processing literature. Accordingly, all powders, including PLA and benzotriazole (C_6_H_5_N_3_), were carefully dried to prevent hydrolytic or thermal degradation. Following reference guidelines [64], 2 g of PLA (10 wt%) was dissolved in 12 mL of chloroform (CHCl_3_), mixed without any heating for at least 1 h under ambient conditions, and sonicated using an ultrasound probe at 20 kHz and 750 W, with pulses of 20 s followed by 5 s of rest, for 30 min, yielding a clear, homogeneous solution with reproducible and controlled viscosity. Functional additives were incorporated sequentially. HDTMS (0.2 mL) was added dropwise to the PLA solution under continuous stirring. The system was kept sealed and stirred at 770 rpm for 30 min until a homogeneous solution was obtained. HDTMS imparts hydrophobicity and modifies the surface energy of the polymer matrix, in agreement with previous reports on silane-modified composites. Epoxidized polybutadiene (EPB), containing randomly distributed epoxy groups along the polymer backbone, was synthesized separately. Following reference guidelines [65], 10 g of PB (C_4_H_6_)*n* was dissolved in 300 mL toluene (C_6_H_5_-CH_3_), mixed with 3.4 g of formic acid at 25 °C, and epoxidized via slow addition of hydrogen peroxide (30 wt% H_2_O_2_) over 15 min. The reaction proceeded for 15 h at 25 °C, after which the organic phase was washed until neutral, precipitated in methanol, and dried under vacuum at 40 °C overnight. The obtained EPB was then incorporated dropwise into the HDTMS-modified PLA solution under continuous stirring to ensure homogeneous dispersion. UV absorbers, typically small organic molecules, were introduced at 0.1–2 wt% of the PLA, following the literature and patent recommendations [66]. Dry benzotriazole (C_6_H_5_N_3_) was added and thoroughly mixed. The resulting mixture was evaluated by torque rheometry at 175–185 °C for 5–10 min to confirm uniform dispersion. After complete incorporation, the solution was diluted, and key quality parameters—including viscosity, fineness, density, and adhesion—were monitored. Finally, the mixture was filtered using sieves or cartridge filters to remove potential impurities, yielding the HDTMS-modified PLA material with incorporated functional additives, suitable for downstream processing and film casting (Table 3: physical parameters of HDTMS-modified PLA solution). After preparation, the formulation was obtained as a liquid resin, whose fluidity is primarily governed by the presence of volatile organic solvents. To preserve its processability, the product was immediately sealed in airtight containers and strictly protected from air exposure, except during controlled use. Premature contact with air leads to rapid solvent evaporation, causing the formulation to solidify and become unsuitable for coating or film formation. Therefore, only the required amount of resin was withdrawn for application, followed by immediate resealing to prevent solvent loss. It is important to distinguish this undesired premature drying from the intended solvent evaporation during film formation. After application onto the perovskite layer, controlled exposure to ambient conditions allows gradual solvent evaporation over approximately 18 h or longer, resulting in the formation of a dense, solid protective film. In this final state, the coating exhibits the desired mechanical integrity and barrier properties, providing effective environmental protection for the underlying perovskite layer.

#### 3.1.3. Monitoring Conditions

**Figure 10:**
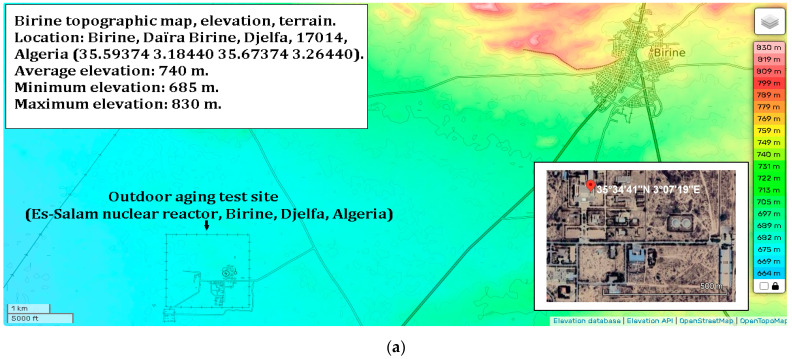

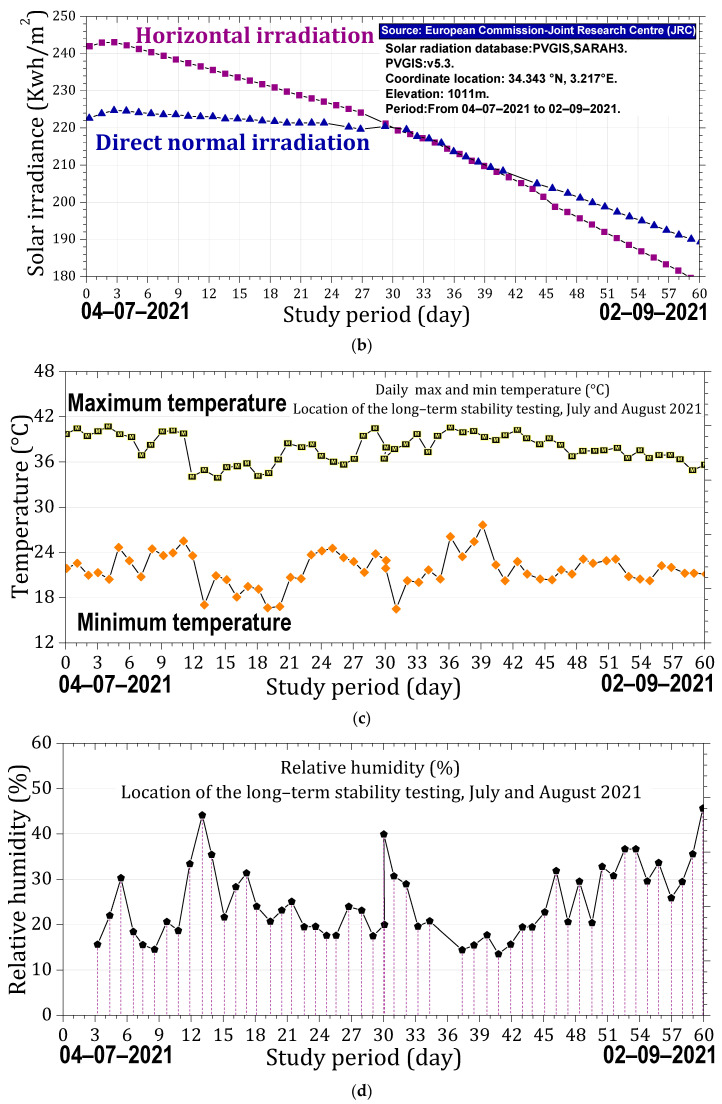

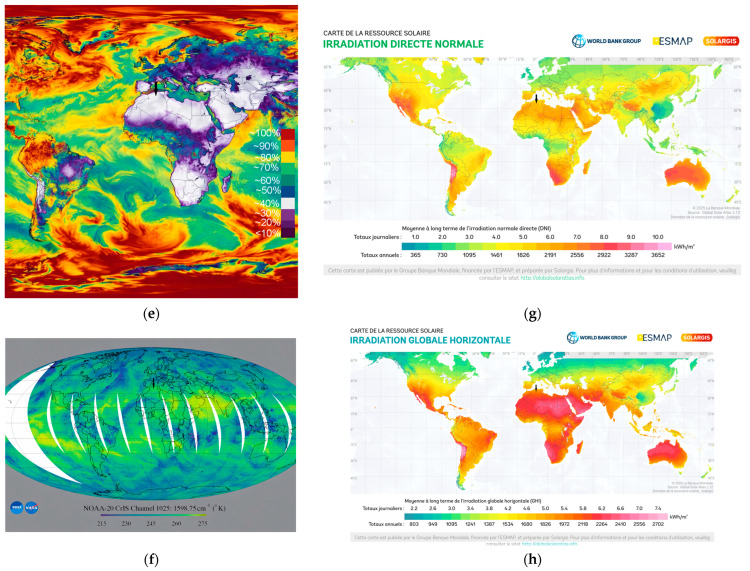
Monitoring conditions of the samples. (**a**) Topographic map of the Es-Salam nuclear reactor site showing elevation above sea level and precise geographical coordinates (latitude and longitude), together with a Google Earth map indicating the reference marker of the exposure location. (**b**) Variation in solar irradiation during the exposure period, including global horizontal irradiation (GHI) and direct normal irradiation (DNI), expressed in kWh·m^−2^. (**c**) Daily maximum and minimum temperature variations recorded throughout the exposure period. (**d**) Relative humidity (%) during the exposure period. Topographic (**e**) Global distribution relative humidity. (**f**) Global distribution of temperature. (**g**) Global distribution of direct solar irradiation. (**h**) Global distribution of horizontal solar irradiation. Arrows indicate the geographical location of the study area in all maps.

The samples were handled using laboratory tweezers to avoid any contact with surfaces that could compromise their integrity. Each sample was placed in an open laboratory glass container, with optical properties similar to the substrate used, and positioned in an open-air laboratory atrium to be exposed to ambient weather conditions. During the day, the samples experienced varying sunlight conditions, at times receiving direct sunlight, at other times partially shaded light; they were predominantly under diffuse light. The stability of the samples was evaluated over a two-month period under the prevailing meteorological conditions at the Es-Salam Nuclear Research Reactor (CRNB), located in the Commune of Birine, Djelfa Province, Algeria (35.58385° N, 3.11746° E), as shown in the topographic map of the Birine area (average elevation ≈ 670 m). The site location was confirmed using an interactive topographic mapping platform (Figure 10a) [67], and an additional site map was also provided. The samples were positioned precisely at this location during the entire exposure period. Meteorological conditions were obtained from the European PVGIS database (Photovoltaic Geographical Information System) for solar irradiation, which provides gridded climatic data worldwide (Figure 10b) [68]. Official regional meteorological records for Djelfa were used for temperature data (Figure 10c) [69] and online weather for relative humidity Figure 10d [70]. Figure 10e presents the global distributions of relative humidity, Figure 10f temperature, and (Figure 10g,h) solar irradiation (direct normal and horizontal). Sample location is indicated with an arrow to facilitate comparison of local meteorological conditions with global patterns. Relative humidity and temperature data were obtained from the Goddard Earth Observing System Forward Processing (GEOS-FP) model, which combines millions of weather observations with predictive modeling to generate a global best estimate of weather conditions (NASA Scientific Visualization Studio) [71]. Global brightness, as an indicator of solar irradiance and cloud cover, was obtained from the CrIS instrument aboard NOAA-20, with dark blue representing liquid water and ice clouds and yellow indicating radiation from the warm Earth’s surface or dry tropospheric layers (STAR CrIS SDR Team) [72]. The detailed data are provided in the Supplementary Materials (Tables S13–S15).

#### 3.1.4. Characterization

To follow-up the degradation of samples, we used X-ray diffraction and UV–Visible spectroscopy techniques. The structural features were performed by X-ray diffraction (XRD) with a diffractometer Philips X’Pert PRO in the Bentano Bragg configuration using the CuKαl emission line with the wavelength λ = 1.5406 Å. The optical properties were studied using UV-Vis with a Shimadzu UV-3101 spectrophotometer at room temperature and in the wavelength range of 300–1200 nm. The initial measurements were first recorded, after which the samples were exposed for a full two-month period under the metrological conditions of the region of Djelfa, a southern department located on the highlands of Algeria. Following this exposure period, the samples were re-measured to evaluate the temporal evolution of their properties.

### 3.2. Methods

#### 3.2.1. Structural Proprieties

The percentages of MAPI and the degradation indicator PbI_2_ were determined by integrating the peak areas of each phase relative to the total spectrum. The texture coefficient $R_{hkl}$ of the film was calculated using (Equation (3)) [73], where $I_{hkl}$ is the experimental diffracted intensity of the (hkl) plane, $I_{0hkl}$ is the intensity from the reference data (JCPDS card No. 96-451-8044), and N is the number of experimental peaks characterizing the phase. The average crystallite size $D_{v}$ was calculated using the Scherrer formula (Equation (4)) [74], where λ is the X-ray wavelength, *θ* is the Bragg angle, β (Equation (5)) is the full width at half maximum FWHM (of perovskite peaks diffraction), and $\beta_{std}$ (Equation (5)) is a correction factor for instrumental broadening. The dislocation density δ was estimated using the (Equation (6)) [75].

(3) $$R_{hkl}=\frac{I_{hkl}/I_{0hkl}}{\frac{1}{N}\sum_{n=1}^{N}\left(I_{hkl}/I_{0hkl}\right)}$$

(4) $$D_{v}=\frac{0.9\;\lambda }{\beta cos\theta }$$

(5) $$\beta =\sqrt{{FWHM}^{2}{{-\beta }_{std}}^{2}}$$

(6) $$\delta =\frac{1}{{D_{v}}^{2}}$$

#### 3.2.2. Optical Properties

For the optical characterizations, the thickness d of our samples was obtained using (Equation (7)) [76], where M is the mass of the thin film, ρ is material density (4.15 gr/cm^3^ for MAPI and 0.9 gr/cm^3^ for the HDTMS-modified PLA as reported by the manufacturer [77]), and a is the surface of the sample. The mass M is obtained from (Equation (8)), where $M_{i}$ is the mass of the substrate and $M_{s}$ is the mass of the sample after deposition of the film [76]. To estimate the band gap energy of the two samples, we used the Tauc model. (Equation (9)) [78]: α is the absorption coefficient, h is the Planck’s constant, ν is the frequency, *n* = 2 for direct allowed transition, A is a constant which is independent of energy, and $E_{g}$ is the band gap energy.

(7) $$d=\frac{M}{\rho a}$$

(8) $$M=(M_{s}-M_{i})$$

(9) $${\left(\alpha h\nu \right)}^{\frac{1}{n}}=A(h\nu -E_{g})$$

## 4. Conclusions

In summary, this study demonstrates that a polylactic-based resin, modified with a hexadecyltrimethoxysilane (HDTMS) formulation, can serve as an effective encapsulation layer, providing exceptional structural and optical stability to highly sensitive MAPI perovskite absorbers under ambient conditions. By developing a methodology that avoids the limitations of conventional approaches such as heat or pressure, which can damage the perovskite layer, this work establishes a practical route for the protective coating of perovskite solar cells. The observed stability can be attributed to the carefully balanced composition of the resin: the predominance of PLA ensures a robust structural matrix, while HDTMS, polybutadiene, and the UV absorber contribute to moisture resistance, flexibility, and photostability of the encapsulation layer. Importantly, the HDTMS-modified PLA encapsulation is not restricted to MAPI but has the potential to be applied across a wide range of photovoltaic technologies, opening new avenues for the integration of environmentally compatible resins in solar cell manufacturing. These findings provide not only a solution to the long-standing challenge of perovskite encapsulation but also lay a robust foundation for future research aimed at enhancing device longevity and environmental resilience, paving the way toward commercially viable, highly stable perovskite-based photovoltaics.

## Figures and Tables

**Table 1:** Summary of XRD peak characteristics: 2θ position, FWHM, d-spacing, instrumental broadening (β*_std_*), peak intensity, and (hkl) planes.

Sample	Peak Position (2θ) (°)	FWHM (°)	β*_std_* (°)	d_hkl_ (A°)	H (Counts)	(hkl)
Initial	11.94	PbI_2_				
	14.33	0.2817	0.11122	6.17477	3721.14	(110)
	20.30	0.09	0.11226	4.37035	97.39	(112)
	21.18	0.2167	0.11278	4.18995	84.28	(200)
	23.64	0.09	0.1133	3.76034	62.37	(211)
	24.31	0.09	0.1133	3.65757	68.37	(202)
	27.53	0.26	0.11382	3.23665	65.47	(004)
	28.68	0.2383	0.11382	3.10911	1721.03	(220)
	30.84	0.3467	0.11435	2.89629	32.77	(213)
	32.06	0.1733	0.11435	2.7892	106.55	(310)
	33.51	0.3467	0.11487	2.67157	37.53	(204)
	40.73	0.3467	0.11695	2.21307	75.41	(400)
	43.56	0.0625	0.11747	2.07578	68.05	(330)
	59.21	0.52	0.11878	1.55924	29.74	(440)
2M-Control	12.97	PbI_2_				
	25.70					
	28.53	0.4093	0.11435	3.1284	105.86	(220)
	38.97	PbI_2_				
	52.64					
2M-PLA	11.99	PbI_2_				
	14.35	0.312	0.11122	6.16344	904.52	(110)
	21.17	0.2184	0.11278	4.19332	163.99	(200)
	23.76	0.0624	0.1133	3.74134	1.28	(211)
	24.36	0.2496	0.1133	3.64982	37.07	(202)
	27.40	0.0936	0.11382	3.25144	104.31	(004)
	28.68	0.5928	0.11382	3.11011	1383.3	(220)
	30.71	0.1872	0.11435	2.90877	40.25	(213)

**Table 2:** Structural parameters of MAPI samples: phase percentages, average crystallite size, and dislocation density for initial analysis, 2-month control, and encapsulated samples.

Sample	MAPI Phase Percentage (%)	PbI_2_ Phase Percentage (%)	The Grain Size D_v_ (nm)	The Dislocation Density δ (10 ^15^ m^−2^)
Initial	98.77	1.23	35.62	0.78
2M-Control	4.11	95.89	21.79	2.10
2M-PLA	96.76	3.24	31.69	0.99

**Table 3:** Physical properties of the commercial HDTMS-modified PLA.

Property	Specification
Physical state	Liquid, clear, glossy
Density	0.902 ± 0.050 g/cm^3^
Viscosity	(Ford Cup 4, 20–25 °C) 60 ± 5 s (~0.07 Pa·s)
Drying time	Surface dry: 6–7 h; Full dry: 24–36 h
Storage	12 months, sealed

## Data Availability

The original contributions presented in this study are included in the article and Supplementary Materials. Further inquiries can be directed to the corresponding author.

## Supplementary Materials

The following supporting information can be downloaded at: https://www.mdpi.com/article/10.3390/molecules31030427/s1, Table S1. Transmittance data of UV–visible spectra of the HDTMS-modified PLA and EVA. Table S2. Absoptance data of UV–visible spectra of the HDTMS-modified PLA and EVA. Table S3. Experimental data for Fourier transform infrared spectroscopy (FTIR) of the HDTMS-modified PLA deposited on a glass substrate, pure PLA, and glass substrate. Table S4. Experimental data for X-ray diffraction (XRD) patterns of MAPI samples without HDTMS-modified PLA encapsulation. Table S5. Experimental data for X-ray diffraction (XRD) patterns of MAPI samples with HDTMS-modified PLA encapsulation. Table S6. Experimental data of pie chart for MAPI and PbI_2_ phases in initial analysis. Table S7. Experimental data of pie chart for MAPI and PbI_2_ phases 2 months without HDTMS-modified PLA. Table S8. Experimental data of pie chart for MAPI and PbI_2_ phases 2 months with HDTMS-modified PLA. Table S9. Experimental data for the evolution of crystallite size of MAPI with (2M-PLA) and without (2M-control) PLA encapsulation over 2 months of environmental exposure. Table S10. Evolution of dislocation density of MAPI with (2M-PLA) and without (2M-control) PLA encapsulation over 2 months of environmental exposure. Table S11. Experimental data for the absorbance spectra of the samples for initial analysis and (without/with) HDTMS-modified PLA after two months of exposure. Table S12. Experimental data for Tauc plots of samples: initial analysis and after two months of exposure (without/with HDTMS-modified PLA). Table S13. Data for the variation in solar irradiation during the exposure period, including global horizontal irradiation (GHI) and direct normal irradiation (DNI). Table S14. Data for daily maximum and minimum temperature variations recorded throughout the exposure period. Table S15. Data for relative humidity (%) during the exposure period.

## Abbreviations

The following abbreviations are used in this manuscript:

MAI	Methylammonium iodide (powder)
MAPI	Methylammonium lead iodide (perovskite CH_3_NH_3_PbI_3_)
HDTMS-modified PLA	A polylactic acid (PLA)-based resin modified with hexadecyltrimethoxysilane (HDTMS)
Initial	First analysis
2M-Control	After 2 months of exposure without HDTMS-modified PLA encapsulation
2M-PLA	After 2 months of exposure with HDTMS-modified PLA encapsulation

## References

[1] Aitola K., Sonai G.G., Markkanen M., Kaschuk J.J., Hou X., Miettunen K., Lund P.D. (2022). Encapsulation of commercial and emerging solar cells with focus on perovskite solar cells. Sol. Energy.

[2] Kojima A., Teshima K., Shirai Y., Miyasaka T. (2009). Organometal Halide Perovskites as Visible-Light Sensitizers for Photovoltaic Cells. J. Am. Chem. Soc..

[3] Miyasaka T. (2018). Development of organic inorganic perovskite high performance solar cells. Impact.

[4] Burschka J., Pellet N., Moon S.-J., Humphry-Baker R., Gao P., Nazeeruddin M.K., Grätzel M. (2013). Sequential deposition as a route to high-performance perovskite-sensitized solar cells. Nature.

[5] Zhou H., Chen Q., Li G., Luo S., Song T.-B., Duan H.-S., Hong Z., You J., Liu Y., Yang Y. (2014). Interface engineering of highly efficient perovskite solar cells. Science.

[6] Kim J.Y., Lee J.-W., Jung H.S., Shin H., Park N.-G. (2020). High-Efficiency Perovskite Solar Cells. Chem. Rev..

[7] Rong Y., Liu L., Mei A., Li X., Han H. (2015). Beyond Efficiency: The Challenge of Stability in Mesoscopic Perovskite Solar Cells. Adv. Energy Mater..

[8] Niu G., Guo X., Wang L. (2015). Review of recent progress in chemical stability of perovskite solar cells. J. Mater. Chem. A.

[9] Berhe T.A., Su W.-N., Chen C.-H., Pan C.-J., Cheng J.-H., Chen H.-M., Tsai M.-C., Chen L.-Y., Dubale A.A., Hwang B.-J. (2016). Organometal halide perovskite solar cells: Degradation and stability. Energy Environ. Sci..

[10] Manser J.S., Saidaminov M.I., Christians J.A., Bakr O.M., Kamat P.V. (2016). Making and Breaking of Lead Halide Perovskites. Acc. Chem. Res..

[11] Bisquert J., Juarez-Perez E.J. (2019). The Causes of Degradation of Perovskite Solar Cells. J. Phys. Chem. Lett..

[12] Kundu S., Kelly T.L. (2020). In situ studies of the degradation mechanisms of perovskite solar cells. EcoMat.

[13] Deretzis I., Alberti A., Pellegrino G., Smecca E., Giannazzo F., Sakai N., Miyasaka T., La Magna A. (2015). Atomistic origins of CH_3_NH_3_PbI_3_ degradation to PbI_2_ in vacuum. Appl. Phys. Lett..

[14] Philippe B., Park B.-W., Lindblad R., Oscarsson J., Ahmadi S., Johansson E.M.J., Rensmo H. (2015). Chemical and Electronic Structure Characterization of Lead Halide Perovskites and Stability Behavior under Different Exposures—A Photoelectron Spectroscopy Investigation. Chem. Mater..

[15] Raga S.R., Jung M.-C., Lee M.V., Leyden M.R., Kato Y., Qi Y. (2015). Influence of air annealing on high efficiency planar structure perovskite solar cells. Chem. Mater..

[16] Yang J., Kelly T.L. (2017). Decomposition and Cell Failure Mechanisms in Lead Halide Perovskite Solar Cells. Inorg. Chem..

[17] Butt M.T.Z., Hussain S.Z., Li X., Briscoe J., Rehman H.U. (2021). Ambient Air-Stable CH_3_NH_3_PbI_3_ Perovskite Solar Cells Using Dibutylethanolamine as a Morphology Controller. ACS Appl. Energy Mater..

[18] Zhong M., Chai L., Wang Y., Di J. (2021). Enhanced efficiency and stability of perovskite solar cell by adding polymer mixture in perovskite photoactive layer. J. Alloys Compd..

[19] Ghosh S., Singh T. (2022). Long Term Stability Assessment of Perovskite Solar Cell Via Recycling of Metal Contacts Under Ambient Conditions. Mater. Lett..

[20] Barichello J., Amiri P., Bellani S., Anichini C., Zappia M.I., Gabatel L., Matteocci F. (2025). Beneath the Surface: Investigating Perovskite Solar Cells Under Water. Energy Environ. Mater..

[21] Wang Y., Ahmad I., Leung T., Lin J., Chen W., Liu F., Ng A.M.C., Zhang Y., Djurišić A.B. (2022). Encapsulation and Stability Testing of Perovskite Solar Cells for Real Life Applications. ACS Mater. Au.

[22] Raman R.K., Gurusamy Thangavelu S.A., Venkataraj S., Krishnamoorthy A. (2021). Materials, methods and strategies for encapsulation of perovskite solar cells: From past to present. Renew. Sustain. Energy Rev..

[23] Wang T., Yang J., Cao Q., Pu X., Li Y., Chen H., Zhao J., Zhang Y., Chen X., Li X. (2023). Room temperature nondestructive encapsulation via self-crosslinked fluorosilicone polymer enables damp heat-stable sustainable perovskite solar cells. Nat. Commun..

[24] Pescetelli S., Agresti A., Viskadouros G., Razza S., Rogdakis K., Kalogerakis I., Spiliarotis E., Leonardi E., Mariani P., Sorbello L. (2022). Integration of two-dimensional materials-based perovskite solar panels into a stand-alone solar farm. Nat. Energy.

[25] Gaddam S.K., Pothu R., Boddula R. (2021). Advanced polymer encapsulates for photovoltaic devices—A review. J. Mater..

[26] Oliveira M.C.C.D., Diniz Cardoso A.S.A., Viana M.M., Lins V.D.F.C. (2018). The causes and effects of degradation of encapsulant ethylene vinyl acetate copolymer (EVA) in crystalline silicon photovoltaic modules: A review. Renew. Sustain. Energy Rev..

[27] Qi Y., Qu J., Moore J., Gollinger K., Shrestha N., Zhao Y., Pradhan N., Tang J., Dai Q. (2022). Interfacial passivation by polylactic acid in perovskite solar cells. Org. Electron..

[28] Xiao H., Zuo C., Yan K., Jin Z., Cheng Y., Tian H., Xiao Z., Liu F., Ding Y., Ding L. (2023). Highly efficient and air-stable inorganic perovskite solar cells enabled by polylactic acid modification. Adv. Energy Mater..

[29] Tripathi N., Misra M., Mohanty A.K. (2021). Durable Polylactic Acid (PLA)-Based Sustainable Engineered Blends and Biocomposites: Recent Developments, Challenges, and Opportunities. ACS Eng. Au.

[30] Wu D., Wessel P., Zhu J., Montiel-Chicharro D., Betts T.R., Mordvinkin A., Gottschalg R. (2023). Influence of Lamination Conditions of EVA Encapsulation on Photovoltaic Module Durability. Materials.

[31] Perpiglia A., Kolb G., Steinfeld A. High and Low Concentrator Systems for Solar Electric Applications IV. Proceedings of the SPIE Solar Energy + Technology 2009.

[32] Moldovan A., Cuc S., Prodan D., Rusu M., Popa D., Taut A.C., Petean I., Bombo¸s D., Doukeh R., Nemes O. (2023). Development and Characterization of Polylactic Acid (PLA) Based Nanocomposites Used for Food Packaging. Polymers.

[33] Jamshidian M., Tehrany E.A., Imran M., Jacquot M., Desobry S. (2010). Poly-Lactic Acid: Production, Applications, Nanocomposites, and Release Studies. Compr. Rev. Food Sci. Food Saf..

[34] Tsuji H., Ikada Y. (1995). Properties and morphology of poly(L-lactide): 1. Annealing condition effects on properties and morphologies of poly(L-lactide). Polymer.

[35] Auras R., Harte B., Selke S. (2004). An overview of polylactides as packaging materials. Macromol. Biosci..

[36] Suwannamek N., Srikulkit K. (2015). Properties of binary and ternary composites of polypropylene containing soybean oil-g-chitosan and hydrophobic phosphonated silica. J. Met. Mater. Miner..

[37] Yazdani S., Hatami M., Vahdat S.M. (2014). The chemistry concerned with the sonochemical-assisted synthesis of CeO_2_/poly(amic acid) nanocomposites. Turk. J. Chem..

[38] Xu B., Zhang Q. (2021). Preparation and Properties of Hydrophobically Modified Nano-SiO_2_ with Hexadecyltrimethoxysilane. ACS Omega.

[39] Dokken K.M. (2006). Infrared Microspectroscopy of Plants: Use of Synchrotron Radiation Infrared Microspectroscopy to Study Plant Root Anatomy and to Monitor the Fate of Organic Contaminants in Those Roots. Ph.D. Thesis.

[40] Mohan S., Settu K. (1993). Vibrational spectra and analysis of 1,2,3-benzotriazole. Indian J. Pure Appl. Phys..

[41] Bodîrlău R., Teacă C.A., Spiridon I. (2009). Composites with PVC & hardwood. BioResources.

[42] Aliberti F., Oliviero M., Longo R., Guadagno L., Sorrentino A. (2025). Effect of crystallinity on the printability of poly(ethylene terephthalate) (PET) and poly(butylene terephthalate) (PBT) blends. Polymers.

[43] Wang F., Chang L., Hu Y., Wu G., Liu H. (2019). Synthesis and Properties of In Situ Bulk High Impact Polystyrene Toughened by High cis 1,4 Polybutadiene. Polymers.

[44] Huang W., Manser J.S., Kamat P.V., Ptasinska S. (2015). Evolution of Chemical Composition, Morphology, and Photovoltaic Efficiency of CH_3_NH_3_PbI_3_ Perovskite under Ambient Conditions. Chem. Mater..

[45] Li Y., Xu X., Wang C., Wang C., Xie F., Gao Y. (2019). Degradation by Exposure of Co-Evaporated CH_3_NH_3_PbI_3_ Thin Films. J. Phys. Chem. C.

[46] Singh P.K., Singh R., Singh V., Tomar S.K., Bhattacharya B., Khan Z.H. (2017). Effect of crystal and powder of CH_3_NH_3_I on the CH_3_NH_3_PbI_3_ based perovskite sensitized solar cell. Mater. Res. Bull..

[47] Misra R.K., Aharon S., Li B., Mogilyansky D., Visoly-Fisher I., Etgar L., Katz E.A. (2015). Temperature- and component-dependent degradation of perovskite photovoltaic materials under concentrated sunlight. J. Phys. Chem. Lett..

[48] Yan J., Song X., Chen Y., Zhang Y. (2020). Gradient Band Gap Perovskite Films with Multiple Photoluminescence Peaks. Opt. Mater..

[49] Fan P., Gu D., Liang G.-X., Luo J.-T., Chen J.-L., Zheng Z.-H., Zhang D.-P. (2016). High-performance perovskite CH_3_NH_3_PbI_3_ thin films for solar cells prepared by single-source physical vapour deposition. Sci. Rep..

[50] Miah M.H., Khandaker M.U., Rahman M.B., Nur-E-Alam M., Islam M.A. (2024). Band Gap Tuning of Perovskite Solar Cells for Enhancing the Efficiency and Stability: Issues and Prospects. RSC Adv..

[51] Costa J.C.S., Azevedo J., Araújo J.P., Santos L.M.N.B.F., Mendes A. (2018). High purity and crystalline thin films of methylammonium lead iodide perovskites by a vapor deposition approach. Thin Solid Film..

[52] Costa J.C.S., Azevedo J., Santos L.M.N.B.F., Mendes A. (2017). On the Deposition of Lead Halide Perovskite Precursors by Physical Vapor Method. J. Phys. Chem. C.

[53] Shirayama M., Kato M., Miyadera T., Sugita T., Fujiseki T., Hara S., Kadowaki K., Murata D., Chikamatsu M., Fujiwara H. (2015). Degradation Mechanism of CH_3_NH_3_PbI_3_ Perovskite Materials upon Exposure to Humid Air. J. Phys. Chem. Lett..

[54] Bouich A., Marí-Guaita J., Baig F., Hameed Khattak Y., Marí Soucase B., Palacios P. (2025). Investigation of the Surface Coating, Humidity Degradation, and Recovery of Perovskite Film Phase for Solar Cell Applications. Sol. Energy Mater. Sol. Cells.

[55] Liu R., Yu R., Tan Z. (2024). Photostability of Perovskite Thin Films under Environmental Stress. Chemistry.

[56] Wu S., Li C., Lien S.Y., Gao P. (2024). Temperature Matters: Enhancing Performance and Stability of Perovskite Solar Cells through Advanced Annealing Methods. Chemistry.

[57] Meng Q., Chen Y., Xiao Y.Y., Sun J., Zhang X., Han C.B., Gao H., Zhang Y.Z., Yan H. (2021). Effect of Temperature on the Performance of Perovskite Solar Cells. J. Mater. Sci. Mater. Electron..

[58] Boro B., Mishra S., Singh T., Lahiri B., Varshney S.K. (2024). Thermal Stability Analysis of Formamidinium–Cesium Based Lead Halide Perovskite Solar Cells Fabricated under Air Ambient Conditions. Energy Technol..

[59] Annohene G., Tepper G. (2021). Moisture Stability of Perovskite Solar Cells Processed in Supercritical Carbon Dioxide. Molecules.

[60] Yang Y., Zhao J., Yang H., Yang X., Lu Y., Huang Z., Duo S., Xiong Z., Hu X. (2025). Green Encapsulants Boost Stability and Sustainability in Inverted Perovskite Solar Cells. Nat. Commun..

[61] Zhang Y.Y., Chen S., Xu P., Xiang H., Gong X.-G., Walsh A., Wei S.-H. (2015). Intrinsic Instability of the Hybrid Halide Perovskite Semiconductor CH_3_NH_3_PbI_3_. arXiv.

[62] Mohammed S.I., Shanshool H.M., Imhan K.I. (2019). Effect of doped Zn–PbI_2_ nanostructures on structural and electrical properties of photodetector applications. J. Theor. Appl. Phys..

[63] Huang H., Chen X., Huang K. (2019). Preparation of PbI2 microflakes by pH-controlled double-jet precipitation. Open Chem. J..

[64] Rajakaruna A.D.N.V., Subeshan B., Asmatulu E. (2022). Fabrication of hydrophobic PLA filaments for additive manufacturing. J. Mater. Sci..

[65] Wang Y., Wei Z., Leng X., Shen K., Li Y. (2016). Highly toughened polylactide with epoxidized polybutadiene by in-situ reactive compatibilization. Polymer.

[66] Zhang X., Li Y., Wang H. Method for Preparing Modified Polylactic Acid. CN104592135A, 28 January 2015. https://patents.google.com/patent/CN104592135A/en.

[67] Topographic Map of Birine (Djelfa Province, Algeria). https://en-gb.topographic-map.com/map-z15ftj/Birine/?utm&center=35.60902,3.16509&zoom=13&base=2.

[68] Photovoltaic Geographical Information System (PVGIS). European Commission Joint Research Centre (JRC). https://re.jrc.ec.europa.eu/pvg_tools/en/tools.html#.

[69] Infoclimat Climatologie de l’année 2021 à Djelfa. https://www.infoclimat.fr/climatologie/annee/2021/djelfa/valeurs/60535.html.

[70] WeatherOnline City Weather Maps and Climatic Data. https://www.weatheronline.co.uk/weather/maps/city.

[71] NASA Earthdata Contributions of NASA’s AIRS Instrument to Global Temperature and Humidity Monitoring. NASA Earthdata. https://www.earthdata.nasa.gov/topics/atmosphere/humidity.

[72] Global Solar Atlas. World Solar Resource Data. https://globalsolaratlas.info/download/world.

[73] Sami A., David E., Fréchette M. Procedure for Evaluating the Crystallinity from X-Ray Diffraction Scans of High and Low Density Polyethylene/SiO_2_ Composites. Proceedings of the Annual Report Conference on Electrical Insulation and Dielectric Phenomena (CEIDP).

[74] Mat Yunin M.Y.A., Mohd Adenam N., Khairul W.M., Yusoff A.H., Adli H.K. (2022). Effect of stability of two-dimensional (2D) aminoethyl methacrylate perovskite using lead-based materials for ammonia gas sensor application. Polymers.

[75] Fassl P., Zakharko Y., Falk L.M., Goetz K., Paulus F., Taylor A., Zaumseil J., Vaynzof Y. (2021). Effect of Density of Surface Defects on Photoluminescence Properties in MAPbI_3_ Perovskite Films. J. Phys. Chem. C.

[76] Kemerchou I., Khechekhouche A., Timoumi A., Rogti F., Hima A., Sadoun A., Tliba A., Aida M.S. (2021). Study of the chemical structure of CH_3_NH_3_PbI_3_ perovskite films deposited on different substrates. J. Mater. Sci. Mater. Electron..

[77] ENAP Product Information: PLA-Based Resin. https://www.enap.dz/fr/detailprod.php?prod=414101000.

[78] Jubu P.R., Yam F.K., Igba V.M., Beh K.P. (2020). Tauc-plot scale and extrapolation effect on bandgap estimation from UV–vis–NIR data—A case study of β-Ga_2_O_3_. J. Solid State Chem..

